# App Designs and Interactive Features to Increase mHealth Adoption: User Expectation Survey and Experiment

**DOI:** 10.2196/29815

**Published:** 2021-11-04

**Authors:** Allison J Lazard, J Scott Babwah Brennen, Stephanie P Belina

**Affiliations:** 1 Hussman School of Journalism and Media University of North Carolina at Chapel Hill Chapel Hill, NC United States; 2 Lineberger Comprehensive Cancer Center University of North Carolina at Chapel Hill Chapel Hill, NC United States; 3 Center on Science and Technology Policy Duke University Durham, NC United States

**Keywords:** smartphone, interactive design, mobile apps, preventive health, mental models, prototypicality, attention, affordances

## Abstract

**Background:**

Despite the ubiquity of smartphones, there is little guidance for how to design mobile health apps to increase use. Specifically, knowing what features users expect, grab their attention, encourage use (via predicted use or through positive app evaluations), and signal beneficial action possibilities can guide and focus app development efforts.

**Objective:**

We investigated what features users expect and how the design (prototypicality) impacts app adoption.

**Methods:**

In a web-based survey, we elicited expectations, including presence and placement, for 12 app features. Thereafter, participants (n=462) viewed 2 health apps (high prototypicality similar to top downloaded apps vs low prototypicality similar to research interventions) and reported willingness to download, attention, and predicted use of app features. Participants rated both apps (high and low) for aesthetics, ease of use, usefulness, perceived affordances, and intentions to use.

**Results:**

Most participants (425/462, 92%) expected features for navigation or personal settings (eg, menu) in specific regions (eg, top corners). Features with summary graphs or statics were also expected by many (395-396 of 462, 86%), with a center placement expectation. A feature to “share with friends” was least expected among participants (203/462, 44%). Features fell into 4 unique categories based on attention and predicted use, including *essential features* with high (>50% or >231 of 462) predicted use and attention (eg, calorie trackers), *flashy features* with high attention but lower predicted use (eg, links to specific diets), *functional features* with modest attention and low use (eg, settings), and *mundane features* with low attention and use (eg, discover tabs). When given a choice, 347 of 462 (75%) participants would download the high-prototypicality app. High prototypicality apps (vs low) led to greater aesthetics, ease of use, usefulness, and intentions, (for all, *P*<.001). Participants thought that high prototypicality apps had more perceived affordances.

**Conclusions:**

Intervention designs that fail to meet a threshold of mHealth expectations will be dismissed as less usable or beneficial. Individuals who download health apps have shared expectations for features that should be there, as well as where these features should appear. Meeting these expectations can improve app evaluations and encourage use. Our typology should guide presence and placement of expected app features to signal value and increase use to impact preventive health behaviors. Features that will likely be used and are attention-worthy—essential, flashy, and functional—should be prioritized during app development.

## Introduction

### Background

With the rapid increase in the use of mobile technologies and smartphones for health information [1,2], mobile apps present one possible solution for communicating preventive health information to the public [3-5]. Over the past decade, hundreds of health mobile apps have been produced—many designed by public health interventionists and researchers for cancer and other chronic disease prevention by encouraging healthy eating and physical activity [6-8]. While it remains unclear how successful these apps have been in reducing the incidence of cancer or improving health outcomes for other chronic diseases, there is a call for an increase in the accountability, reliability, and standardizations of evidence-based health apps developed by the research community [8-10].

Despite the potential of mobile health (mHealth) apps for communicating up-to-date, evidence-based prevention information and helping users maintain or implement healthy habits, there is very little guidance on how these intervention apps should be designed to ensure adoption [11]. Designing apps so they are appealing and used is a critical first step for apps to have an impact [12]. Visual and interactive design influences initial user evaluations, which are made within milliseconds, and serve as gateways for subsequent user engagement (eg, use) of apps as mHealth interventions [13-15]. Ignoring design can detrimentally impact the communication of evidence-based science to health consumers and undercut the effectiveness of mHealth interventions; yet, few mHealth interventions mirror the look and function of popular, industry-developed apps. Thus, our study objective was to explore app features expectations and examine how meeting expectations with high- (vs low-) prototypicality apps may influence predictors of app adoption.

How apps are designed (visual display) and the features they include (interactivity) can influence users’ experience of and willingness to engage with apps. Individuals use salient cues that match their expectations, or mental models, to evaluate web-based information [16,17]. These expectations are met (or not) by the level of prototypicality or the degree to which an app resembles others in its comparative group [17,18]. Based on included design cues, in the form of interactive features, apps can range from having high prototypically (looks like others and meets expectations well) to low prototypicality (does not resemble others nor meet expectations) [19]. Users are often quicker and more willing to attend to apps that have high prototypicality—when designs align with one’s mental models for how an app should look and function [19-21]. Indeed, users look for and pay attention to expected, salient features as guides to orient themselves to novel apps and platforms [21]. When these expected features are present, they increase familiarity and potential use of the app [19-21]; however, little is known on how attention for specific features translates into individual feature use versus overall app use.

The perceived affordances, or perceived action possibilities (eg, learn health tips), that users sense from app features also directly impact a user’s experience and likelihood to engage with a design [22-24]. Specifically for mediated communication, including apps, design communicates what the viewer can do or gain from the use of an app, through interface symbols. Thus, not only must mHealth interventions have evidence-based content to drive use, but also apps must incorporate an evidence-based design to appeal to and engage audiences.

Design features influence the appeal or perceived aesthetics of the app and the likelihood for use [25,26]. To be effective, health apps must surely be used. It is necessary to understand how objective design features (the visible objects or designs in an app) influence subjective evaluations for initial appeal on the basis of theories of aesthetics [27-29] and antecedents for technology adoption in the Technology Acceptance Model (TAM); that is, perceived ease of use, perceived usefulness, and intentions to use [30,31]. Aesthetics, including facets for how information is organized and displayed, function as a precursor to perceptions for technology acceptance [28,31]. Accounting for users’ expectations of features and placements within apps will shed light on how prototypicality impacts evaluations critical for future adoption.

Utility also drives evaluation of an app’s usefulness and potential adoption, according to Nielsen et al’s [32] well-established usability study. Utility refers to the inclusion of necessary features—whether an app provides the elements an individual needs or wants. When utility is paired with usability—when features are perceived as easy (perceived ease of use) and pleasant (aesthetics) to use—individuals are encouraged to engage or interact. In other words, interactivity is dependent on a user’s willingness to engage with specific design features, if present (utility) and function properly (usability). In our work, we focus on the former—how app features that are needed (utility) or expected (prototypical) are the gateway to potential adoption.

### Goal of This Study

In sum, engagement with and use of an app is driven by initial impressions and perceptions of what the app can do for the user. Top-rated industry-developed apps often incorporate a user-focused sleekness and are feature loaded; in comparison, pared-down mHealth interventions—despite the inclusion of theory-based content—may not appeal to audiences who need them [33]. When resources are not abundant, health researchers and interventionists need evidence-based guidance for design investments. Thus, we explored app expectations for the presence and placement of potential features, how these features garner attention and predict use, and how high-prototypicality apps (vs low-prototypicality apps) may influence app adoption through app choice and predictors of use. We asked the following research questions: What features do people expect and where do they expect these features to be placed (RQ1)? What specific features are associated with attention and predicted use of the app features (RQ2)? Last, we also examined whether high prototypicality, resembling that of top downloaded apps (vs low-prototypicality apps, resembling research intervention apps) would increase app choice (H1), aesthetics (H2), perceived ease of use (H3), perceived usefulness (H4), intentions to use the app (H5), and perceived affordances or action possibilities with the app (H6).

## Methods

### Overview

To explore app features expectations and examine how meeting expectations with high-prototypicality apps (vs low-prototypicality apps) may influence predictors of app adoption, we conducted a web-based survey with an embedded within-subjects experiment. Participants first responded to survey items about expectations for specific app features to answer RQ1-2 and an app choice (preview of apps with high vs low prototypicality) to address H1. Participants were then asked to rate their perceptions of the app overall, with the exposure order of condition (high vs low) randomized, to address H2-6.

### Participant Recruitment

Using G*Power, our a priori power analysis indicated a required sample of at least 450 participants to detect a small-to-medium effect (Cohen *f*=0.14) for within-subjects comparison of the high and low prototypicality apps. Participants (n=462) were recruited from Amazon’s Mechanical Turk (MTurk), a web-based crowdsourcing platform often used for social science research [34-36], through a link open to individuals over the age of 18 years. Participants were eligible if they were aged 18 years or older, resided in the United States, and had a task approval rate of 85% or higher on the MTurk platform, which indicates valid participation or completion of previous tasks. Participants received US $3 as compensation for their time (approximately 15 minutes). The institutional review board of University of North Carolina approved this study.

### Procedure

Following consent, participants selected features (from a list) they would expect to find in a health app. For all expected features, participants were shown an outline of a smartphone and asked where that feature would be located in a typical health app. Participants were then randomly assigned to 1 of the 2 app types for the remainder of the study: fitness apps or nutrition apps. Participants selected the app they would most like to download from 2 previews (prototypical: high vs low). On subsequent pages, participants indicated what features grabbed their attention and what features they predicted they would use (predicted use) on their preferred app. Participants were shown the app previews again (one at a time, in a random order) and asked closed-ended items for perceived aesthetics, ease of use, usefulness, intentions to use the app in the future, and perceived affordances. Lastly, demographic, health, and health app information were collected from all participants. Closed-ended items and response options are described below (see Measures) and provided in Multimedia Appendix 1.

### App Stimuli

**Figure 1:**
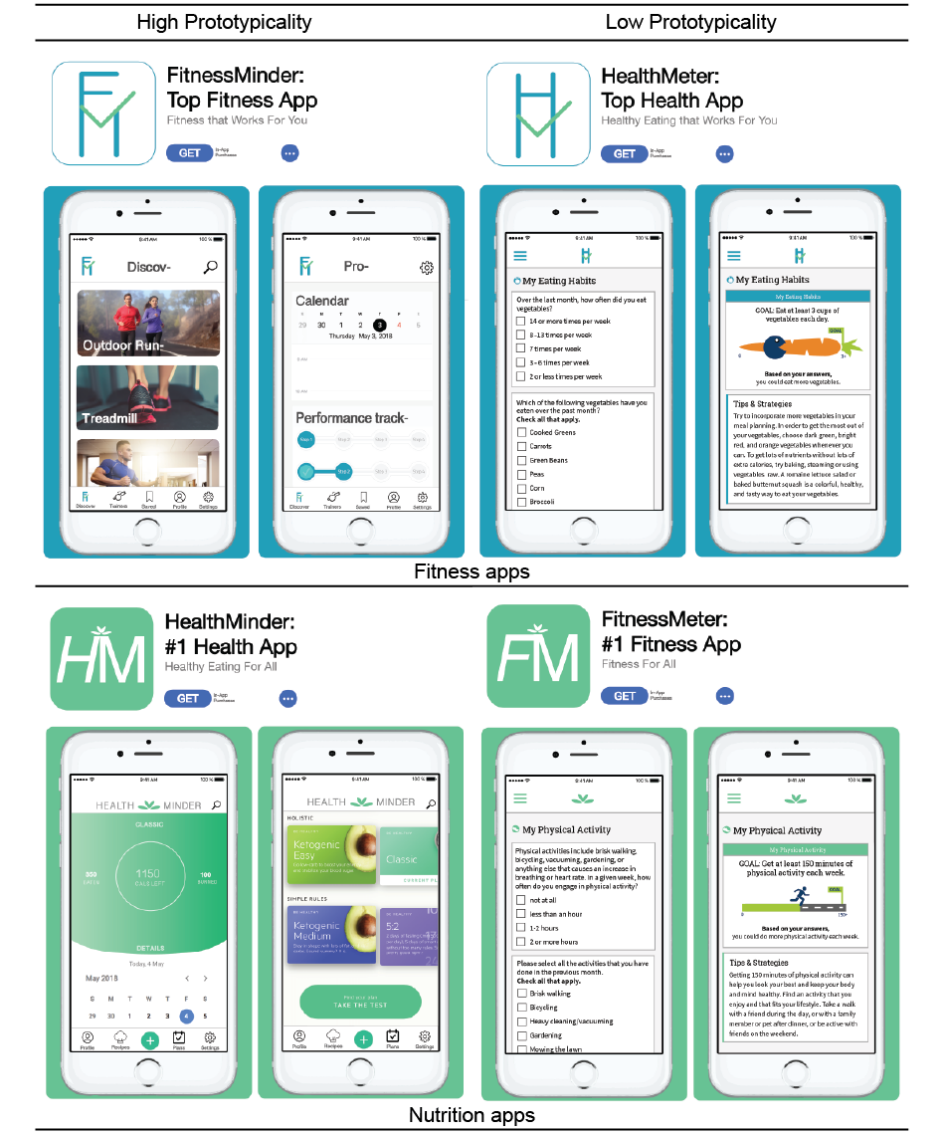
App preview stimuli.

To assess the impact of prototypicality on app perceptions, app previews were created for four fictitious brands: 2 fitness and 2 nutrition health apps (Figure 1). We designed previews for each app as they would appear if searched for in a mobile app store, including the app icon, brand name, and 2 preview screens of the app. High-prototypicality apps were developed on the basis of structure and content from top rated apps (Aaptiv, Lifesum) in the Health & Fitness section of the App Store. Low-prototypicality apps were designed to mirror the mobile interface of an interactive intervention (Carolina Health Assessment and Research Tool) for data collection and tailored feedback for preventive health behaviors [37].

### Measures

#### Feature Selection and Placement

Participants selected features from a list they “would expect to find in a health app.” The list was generated from structured interviews about fitness tracker apps [38] and included 12 features: menu, search option, settings option, logo, log/input data option, share with friend option, summary statistics, summary graph/chart, calendar, page title, login, and user profile. For each expected (ie, selected) feature, respondents were shown a smartphone screen divided into a grid of 60 distinct clickable hot spot regions. Respondents selected as many regions of each screen as necessary for expected placement.

#### App Choice

Participants were instructed to “select the app you would most likely download.” The 2 response options were the low prototypical app and the high prototypical app, for their randomly assigned app type (physical activity or nutrition).

#### Feature Attention and Predicted Use

To identify features that attracted participants’ attention and predicted use, participants were shown the app preview they selected during app choice. Participants were asked, “What elements in the app caught your attention?” and instructed to “select all elements that grabbed your attention within the app preview.” On the following page of the questionnaire the app preview was shown again; participants were asked, “What elements in the app do you think you would use?” and selected the elements in the preview. As performed in previous studies [39,40], a priori hot spots were constructed around each app feature (Multimedia Appendix 1). Hot spots were not visible until participants selected the feature and then the feature was highlighted.

#### Perceived Aesthetics

The validated Visual Aesthetics of Website Inventory (VisAWI) assessed 4 facets of aesthetics with 18 items for simplicity, “The layout appears well structured”; diversity, “The layout appears dynamic”; colorfulness, “The colors are appealing”; and craftsmanship, “The app is designed with care” [28]. Response options ranged from “strongly disagree” (coded as 1) to “strongly agree” (5). Responses were averaged for each facet (α=.76-.90).

#### Perceived Ease of Use

Participants’ perceived ease of use, or belief that using the technology would not be difficult, were assessed with 3 adapted Likert-type items [30]: “The app was clear and understandable,” “Getting the app to function does not require much mental effort,” and “I find the app to be easy to use.” Response options ranged from “strongly disagree” (coded as 1) to “strongly agree” (5). Responses were averaged (α=.84-.87).

#### Perceived Usefulness

The degree to which one believes that the technology will enhance their life was assessed with 3 adapted Likert-type items [30]: “Using the app would improve my health,” “Using the app would make me more likely to meet my health goals,” and “I would find the app useful for achieving my health goals.” Response options ranged from “strongly disagree” (coded as 1) to “strongly agree” (5). Responses were averaged ( =.85-.88).

#### Intentions to Use

Intentions or plans to use the app “if the app were available” were assessed with 2 Likert-type items [30]. Participants rated their agreement to statements that they “intend” and “predict” they would use the app next month with response options that ranged from “strongly disagree” (coded as 1) to “strongly agree” (5). Responses were averaged (*r*=0.88-0.93).

#### Perceived Affordances

Participants reported perceived action possibilities from the app with the item, “This app would allow me to…” Response options included a list of 13 dichotomous items generated from evidence-based behavior change techniques and reasons for eHealth adoption, such as “set health goals,” “track my progress,” “earn rewards,” and “share my health data with friends” [41,42].

#### Participant Characteristics

Demographic items assessed age, gender, race, ethnicity, and education. Additionally, we asked about one’s health and mental health status with the item: “in general, would you say your [mental] health is…” Response options ranged from “very poor” (coded as 1) to “very good” (5). We also asked whether participants “use a health app” with a “yes”/”no” response option.

### Data Analyses

We used n (%) values to describe app feature expectations, placement, app choices, attention, predicted use, and perceived affordances. Frequencies for attention vs predicted use and for perceived affordances of the high- vs low-prototypicality apps were compared with McNemar chi-square tests. Prior to this analysis for direct effects of prototypicality, a multivariate analysis of variance (MANOVA) was used to determine if there are any significant differences in perceptions among the app types (fitness and nutrition) across aesthetics and TAM outcomes. No differences were observed for high prototypicality (aesthetics outcomes: Wilks λ=0.98; *F*_4,454_=1.08; *P*=.10; TAM outcomes: Wilks λ=0.99; *F*_3,454_=1.08; *P*=.36) or low prototypicality (aesthetics outcomes: Wilks λ=0.99; *F*_3,452_=0.68; *P*=.61; TAM outcomes: Wilks λ=1.00; *F*_3,454_=0.10; *P*=.96), so data within conditions (high vs low prototypicality) were combined for analyses. Two repeated measure (RM) MANOVAs and analyses of variance (ANOVAs) were then conducted with high vs low prototypicality as the predictor; 1 for aesthetic outcomes (simplicity, diversity, colorfulness, and craftsmanship) and 1 for technology acceptance outcomes (perceived ease of use, usefulness, and intentions to use).

## Results

### Participants

Participants (n=462) were aged 18 to 70 years (mean age 35.03 years, SD 10.02 years) and half of them were female (50%, 232/462). Participants identified as White (78%, 358/462), African American (13%, 58/462), Asian (8%, 35/462), or multiracial/other; additionally, 48 of 462 participants (10%) reported their ethnicity as Hispanic. Education levels included high school to some college (33%, 153/462), associate degree (13%, 60/462), bachelor’s degree (43%, 197/462), master’s degree (10%, 45/462), and doctoral or professional degree (2%, 7/462). Most participants reported their health as good (48%, 220/462) or very good (17%, 78/462), although some did report that their health was fair (30%, 138/462), poor (4%, 20/462), or very poor (1%, 4/462). Over half of the participants (53%, 248/462) reported currently using health apps.

### App Feature Selection and Placement

Each of the 12 features was selected by at least 44% (203/462) of participants (RQ1). The majority of participants (92%, 425/462) selected a menu, settings options, and user profile; notably, these features (ie, menu, settings option, and user profile) were selected an equal number of times but not by the *same* respondents. Additional features were expected, including the following: login (88%, 406/462), summary graph/chart (86%, 396/462), summary statistics (86%, 395/462), input data feature (80%, 368/462), calendar (77%, 354/462), logo, (77%, 357/462), search (69%, 321/462), page title (62%, 286/462), and an option to “share with friends” (44%, 203/462).

**Figure 2:**
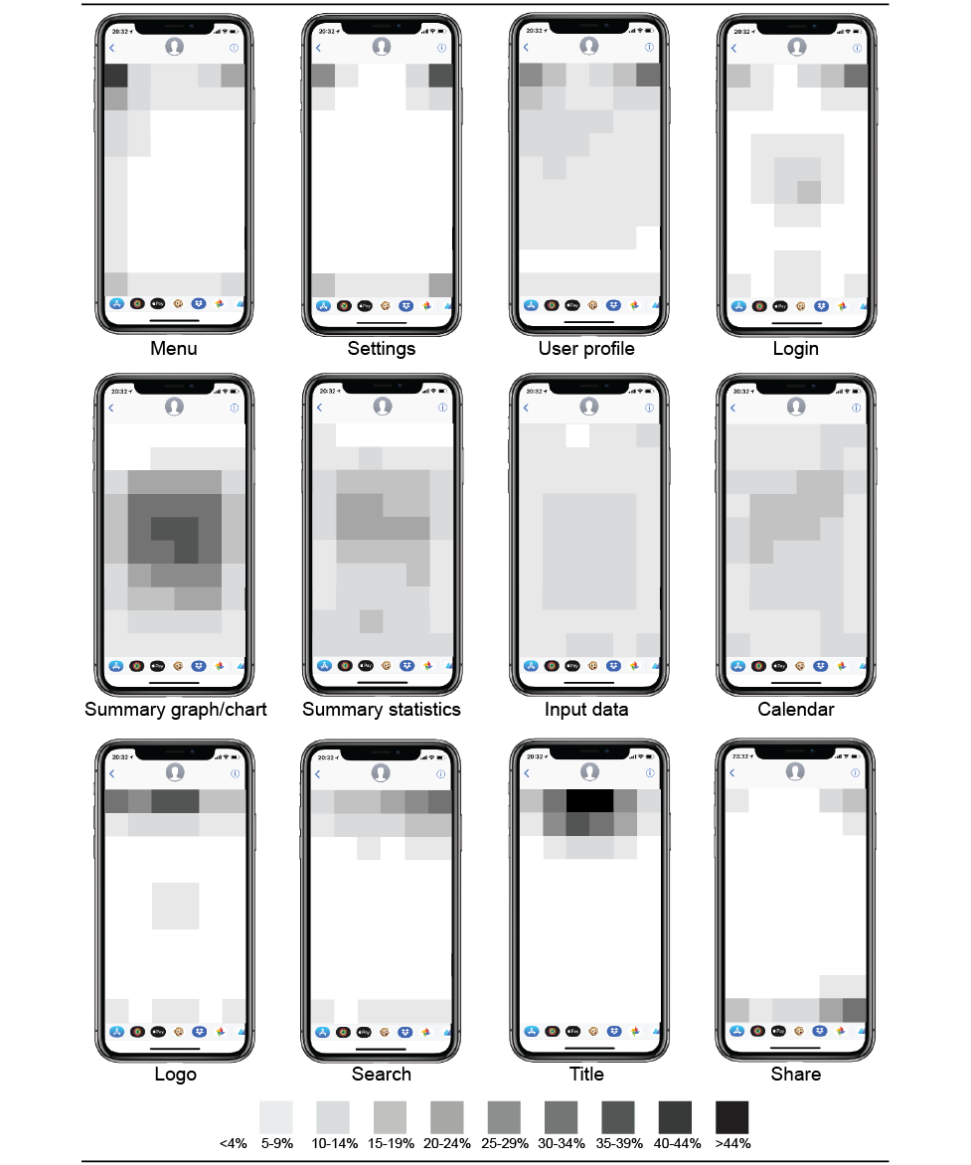
Expected app feature location (n=425).

Most features were expected in similar locations (Figure 2) among participants who had expected features (n=425). Menus were consistently expected to be in the top-left, while search and login options are placed in the top-right corner. Other features—title, logo, profile, and settings—were expected along the top, in the center, or either side. Sharing capability was expected to appear in the bottom-right of the app, although expectations of where to log input data were more diffuse. Users expect summary statistics, graphs, and calendars to be shown across the center of the app.

### Attention and Predicted Use of App Features

Respondents selected features of their preferred app, which caught their attention and they would use (Table 1 and Multimedia Appendix 1). Attention and predicted use patterns of the high-prototypicality apps indicate 4 distinct categories of mHealth app features. *Mundane features* are those that have similar low attention and predicted use values. In the fitness app, the footer menu options “Discover” and “Saved” represent mundane features. *Functional features* have higher predicted use than attention, but predicted use remains low (<50%, <231/462) among participants, such as the settings icon in both apps. *Flashy features* are elements identified as attention-capturing by most participants (>50%, >231/462), and attention is significantly higher than the predicted use. In the nutrition app, large photo-based links for the “Ketogenic Easy” and “Ketogenic Medium” diets represent flashy features. *Essential features* are elements that most participants (>50%, >231/462) thought they would use, and where predicted use is higher than or similar to attention, as with the “Calorie Tracker” in the nutrition app. Not included in these 4 categories are elements that have higher attention than predicted use, but the attention remains low (<50%, <231/462); the only features with these characteristics were logos and app titles, as well as 2 features partially obscured in the design.

**Table 1:** Reported attention and predicted use of app features (n=462).

App		Feature	Attention, n (%)	Predicted use, n (%)	Chi-square (*df*)	*P* value
**Mundane**						
	Fitness	Footer menu option “Discover”	48 (29)	52 (32)	0.20 (1)	.66
	Fitness	Footer menu option “Saved”	41 (25)	42 (26)	0.00 (1)	>.99
	Nutrition	Footer menu option “Plus”	23 (13)	28 (15)	0.46 (1)	.50
**Functional**						
	Fitness	Footer menu option “Settings”	49 (30)	69 (42)	7.22 (1)	.007
	Nutrition	Search Icon	21 (12)	38 (21)	5.95 (1)	.02
	Nutrition	Footer menu option “Profile”	25 (14)	58 (32)	19.32 (1)	<.001
**Flashy**						
	Fitness	Activity 1 “Outdoor Running”	109 (66)	63 (38)	32.66 (1)	<.001
	Fitness	Acitivty 2 “Treadmill”	104 (63)	53 (32)	38.46 (1)	<.001
	Nutrition	Ketogenic Easy feature	109 (60)	71 (39)	20.74 (1)	<.001
**Essential**						
	Fitness	Performance Tracker feature	142 (86)	157 (95)	N/A^a^	.003
	Nutrition	Calorie Tracker feature	156 (86)	160 (88)	N/A	.54
	Nutrition	Calendar feature	88 (48)	114 (63)	11.57 (1)	.001

^a^N/A: chi-square values are not applicable if fewer than 25 discordant pairs; binominal distributions are used for exact 2-tailed significance in these comparisons.

### Effects of Prototypicality on App Choice, Aesthetics, and Technology Acceptance

When asked to choose between the high-prototypicality app and one designed to look more like a typical health intervention (low prototypicality), 347 of 462 (75%) participants indicated they would download the high-prototypicality app (H1).

Prototypicality had a significant main effect on all facets of aesthetics and technology acceptance outcomes (Table 2). High-prototypicality apps (vs low-prototypicality apps) had significantly higher ratings of aesthetics for simplicity (*F*_1,455_=291; *P<*.001), diversity (*F*_1,455_=578; *P<*.001), colorfulness (*F*_1,455_=295; *P<*.001), and craftsmanship (*F*_1,455_=462; *P<*.001). Similarly, the high-prototypicality app was rated higher than the low prototypicality app for perceived ease of use (*F*_1,455_=84; *P<*.001), usefulness (*F*_1,455_=116, *P<*.001), and intentions to use the app (*F*_1,455_=170; *P<*.001). H2-5 were supported.

**Table 2:** Main effects of prototypicality on aesthetics and technology acceptance (n=456).

Attributes	High prototypicality, mean (SD)	Low prototypicality, mean (SD)	*F* test (*df*)	*P* value
Simplicity	4.26 (0.74)	3.19 (1.00)	291 (*1,455*)	<.001
Diversity	4.10 (0.74)	2.48 (1.09)	578 (*1,455*)	<.001
Colorfulness	4.38 (0.74)	3.41 (0.94)	295 (*1,455*)	<.001
Craftsmanship	4.25 (0.75)	2.83 (1.07)	462 (*1,455*)	<.001
Perceived ease of use	4.26 (0.75)	3.74 (0.97)	84 (*1,455*)	<.001
Perceived usefulness	4.08 (0.74)	3.58 (0.91)	116 (*1,455*)	<.001
Intentions to use	3.83 (1.00)	2.95 (1.28)	170 (*1,455*)	<.001

### Impact of Prototypicality on Perceived Affordances

Participants reported that the app would allow them to carry out various actions in both the high- and low-prototypicality design (Table 3). Almost all perceived affordances had significantly higher endorsement for the high-prototypicality (vs low-prototypicality) apps (*P*<.01), partially supporting H6; to “learn health tips” was the only affordance endorsed similarly in both conditions. The most highly endorsed affordances (>60% across conditions or >277/462) were the following: “track my progress” (high: 93%, 430/462; low: 70%, 325/462), “set health goals” (high: 88%, 405/462; low: 73%, 339/462), “improve my health” (high: 74%, 342/462; low: 63%, 293/462), “learn health tips” (high: 73%, 336/462; low: 76%, 353/462), and “give me more information about my health” (high: 70%, 325/462; low: 63%, 292/462).

**Table 3:** Frequencies and McNemar chi-square differences for perceived affordances (n=462).

Affordances	High prototypicality, n (%)	Low prototypicality, n (%)	Chi-square (*df*)	*P* value
Track my progress	430 (93.1)	325 (70.3)	79.70 (*1*)	<.001
Set health goals	405 (87.7)	339 (73.4)	30.75 (*1*)	<.001
Improve my health	342 (74.0)	293 (63.4)	20.15 (*1*)	<.001
Learn health tips	336 (72.7)	353 (76.4)	2.59 (*1*)	.11
Give me more information about my health	325 (70.3)	292 (63.2)	6.86 (*1*)	.009
Create new health habits	310 (67.1)	265 (57.4)	11.06 (*1*)	.001
Increase my control over my health	323 (69.9)	239 (51.7)	46.86 (*1*)	<.001
Make meeting my health goals easier	292 (63.2)	195 (42.2)	51.28 (*1*)	<.001
Have fun with technology	256 (55.4)	135 (29.2)	79.57 (*1*)	<.001
Interact with others	120 (26.0)	47 (10.2)	56.63 (*1*)	<.001
Share my health data with friends	100 (21.6)	47 (10.2)	35.12 (*1*)	<.001
Share my health data with a healthcare provider	74 (16.0)	50 (10.8)	11.50 (*1*)	.001
Earn rewards	57 (12.3)	34 (7.4)	10.30 (*1*)	.001

## Discussion

### Principal Findings

For mHealth to have an impact on reducing risk for chronic disease, intervention apps must be designed to effectively reach wide audiences to promote preventive health behaviors. Identifying the impact of prototypicality—the extent to which apps meet expectations—on app reception and adoption is a critical step in mHealth intervention research. Designs that match users’ perceptions of organization and content evoke prototypicality and can influence intentions to use web-based tools, including health resources [21,31,43]. Our study on prototypicality serves as an antecedent to positive app reception and technology acceptance in preventive health apps. We also found designs that contradict what users typically expect from apps (eg, low prototypicality), leading to a suboptimal first impression and diminishing users’ expectations [19].

It is likely that the actual use of multiple apps influences preventive behavior [44]; thus, identifying key features, or classes of features, to increase orientation and facilitate ease of use and usefulness are needed to guide intervention development. Our findings for user attention and predicted use of features point to 4 distinct types of mHealth features that should be considered when developing mHealth. Of these, 3 categories serve as useful features of mHealth: driving attention, perceived use, or both.

Functional features have higher predicted use than attention, and a majority “would expect to find” these sorts of features in a health app. To meet expectations, salient functional features such as search options, settings, and menus should be included, in their expected corner placement. Even if these features do not draw attention as much as others, users still expect to see them in mobile apps, and meeting baseline expectations can reduce time and cognitive demand for initial orientation and web-based information processing [21]. Arguably, these functional features constitute a sort of prototypical milieu or background environment for mHealth apps to likely help users orient themselves within new and unfamiliar apps.

Flashy features garner significantly more attention from users; these attention-capturing features may be most influential for positive initial impressions. Flashy features often incorporated photographs or novel design elements, which have been shown to increase attention and appeal [43,45]. Beyond meeting expectations, flashy features represent the unique category that should be treated *differently* in designs: using visuals to highlight salient benefits and perceived affordances.

Essential features—including those selected by most users as features that they predict to use and garner their attention—are also important components of mHealth designs. It is important to note, however, that the essential features seen in this study are all familiar: calendar, calorie counter, and performance tracker. Even though some designers may assume that features as basic as a calendar are not worth the time and effort to include, respondents strongly indicated that these features remain important components of mHealth apps.

Our findings also highlight a distinct category that can be skipped or given little attention in development: mundane features. Mundane features, such as app title and tabs for discovering or saving, elicited little attention and predicted use and are a good indication not to waste precious resources on these elements.

Potential mHealth users had consistent expectations for some features by region (eg, middle or top corner), but not necessarily a specific location. Essential features, such as a calendar, were expected to be shown across the center of the app. Other features, such as function features including *search* and *settings*, had more narrow placement expectations. Understanding these location expectations is critical to ensure that feature placement matches individual models [21].

Higher prototypicality led to higher ratings for aesthetics, perceived ease of use, usefulness, and intentions to use apps. Individuals also expect greater function, possibilities, and valuable outcomes from apps with higher prototypicality. Low prototypicality led to lower rankings for aesthetics, perceived ease of use, and perceived usefulness. Additionally, low prototypicality runs the risk of users initially dismissing the app. Negative product evaluations—where expectations are not met—can also lower satisfaction with product interaction [46].

### Limitations

This study is limited to the specific health apps manipulated herein; these apps do not represent all available mHealth strategies. Although we evaluated placement, attention, and predicted use, we could have reviewed more features within apps. Our findings are also limited to a convenience sample of participants of a web-based panel. It is possible that our participants have more digital literacy or skills than the general population or diverse subgroups.

### Future Work

Future studies should consider assessing actual use after download, instead of solely predicted use. Replication with more diverse audiences, varied app designs, and expanded methodological approaches are needed to generalize our findings. Notably, future research should account for additional personal characteristics, such as health literacy or the ability to obtain, process, and understand health information [47], to examine how these skills affect both first impressions for app adoption and actual use to determine the effectiveness of health apps.

### Conclusions

Mobile apps can communicate critical health information for preventive health behaviors through readily available and consumer-friendly tools. Apps that are thoughtfully designed to match potential users’ expectations, with increased prototypicality, will support app use. Conversely, designs that do not include a threshold of expected features will be dismissed, thus undermining the potential of app-based interventions. Designing mHealth apps to account for user expectations will increase the likelihood of adoption and impact from actual use. Prototypicality is positively related to favorable reception and expectations for future use of health apps. These findings provide guidance for user expectations of feature presence and location.

## Appendix

Multimedia Appendix 1 App hot spots and survey items.

## Abbreviations

ANOVA: analysis of variance
MANOVA: multivariate analysis of variance
mHealth: mobile health
MTurk: Mechanical Turk
RM: repeated measures
TAM: technology acceptance model
