# Supplementary material for: App Designs and Interactive Features to Increase mHealth Adoption: User Expectation Survey and Experiment
Source: JMIR Mhealth Uhealth. 2021 Nov 4;9(11):e29815. doi: 10.2196/29815 (PMC8603164; doi:10.2196/29815)
Supplement: Multimedia Appendix 1 [file mhealth_v9i11e29815_app1.pdf]

Appendix A. App Preview Hot Spots for Attention and Use

Table A1. Attention and Predicted Use of Features in the High Prototypicality Fitness App (n = 165)

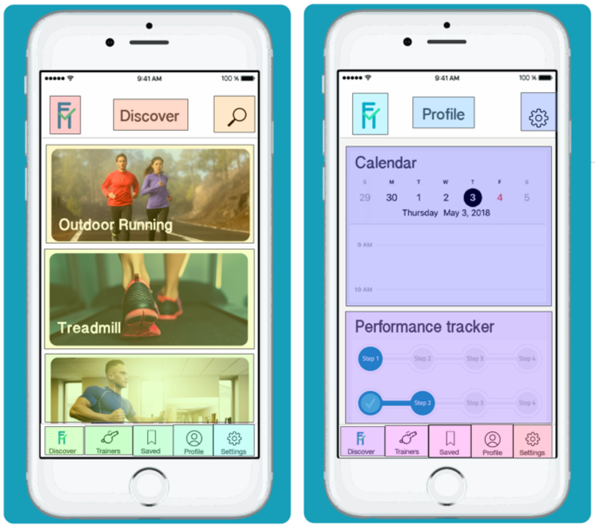

	Attention		Use			
	n	%	*n*	%	χ^2	*P*
Left app preview						
Header app logo icon (top left)	38	23%	14	9%	**17.63**	**<.001**
Header page title (“Discover”)	34	21%	26	16%	n/a	.15
Header search icon	40	24%	57	35%	**7.31**	**.007**
Activity 1 “Outdoor running”	109	66%	63	38%	**32.66**	**<.001**
Activity 2 “Treadmill”	104	63%	53	32%	**38.46**	**<.001**
Activity 3 “Individual Exercising”	90	55%	36	22%	**40.13**	**<.001**
Footer menu option “Discover”	48	29%	52	32%	.20	.66
Footer menu option “Trainers”	49	30%	36	22%	3.35	.07
Footer menu option “Saved”	41	25%	42	26%	.00	1.00
Footer menu option “Profile”	53	32%	70	42%	**5.02**	**.03**
Footer menu option “Settings”	49	30%	69	42%	**7.22**	**.01**
Right app preview						
Header app logo icon (top left)	24	15%	11	7%	n/a	**.02**
Header page title “Profile”	37	22%	32	19%	.49	.49
Header settings icon	46	28%	62	38%	**4.89**	**.03**
Calendar feature	**107**	**65%**	**118**	**72%**	1.96	.16
Performance tracker feature	142	86%	157	95%	**n/a**	**.003**
Footer menu option “Discover”	42	26%	48	29%	.52	.47
Footer menu option “Trainers”	48	29%	39	24%	1.36	.24
Footer menu option “Saved”	40	24%	45	27%	.37	.54
Footer menu option “Profile”	49	30%	75	46%	**12.50**	**<.001**
Footer menu option “Settings”	52	32%	71	43%	**7.20**	**.01**

Note: n/a = chi square values are not applicable if fewer than 25 discordant pairs; binominal distributions are used for exact 2-tailed significance in these comparisons.

Table A2. Attention and Predicted Use of Features in the High Prototypicality Nutrition App (n = 182)

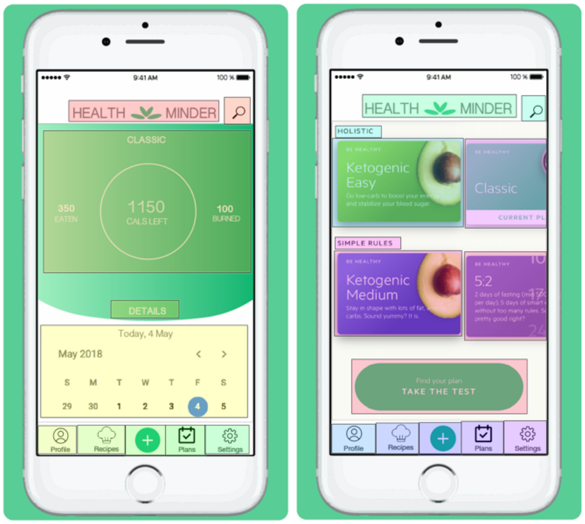

	Attention		Use			
	n	%	*n*	%	χ^2	*P*
(LEFT SIDE)						
Header app logo icon (top middle)	38	21%	8	4%	23.36	**<.001**
Header search icon	19	10%	54	30%	**21.81**	**<.001**
Calorie tracker feature	**156**	**86%**	**160**	**88%**	n/a	.54
Details icon	14	8%	50	28%	**26.63**	**<.001**
Calendar feature	88	48%	114	63%	**11.57**	**.001**
Footer menu option “Profile”	37	20%	90	50%	**38.09**	**<.001**
Footer menu option “Recipes”	58	32%	106	58%	**32.49**	**<.001**
Footer menu option “Plus”	30	17%	45	25%	**4.56**	**.03**
Footer menu option “Plans”	50	28%	102	56%	**36.13**	**<.001**
Footer menu option “Settings”	35	19%	87	48%	**36.13**	**<.001**
(RIGHT SIDE)						
Header app logo icon (top middle)	40	22%	12	7%	**20.25**	**<.001**
Header search icon	21	12%	38	21%	**5.95**	**.02**
Holistic icon	4	2%	9	5%	n/a	.13
Ketogenic Easy feature	109	60%	71	39%	**20.74**	**<.001**
Footer menu option “Profile”	25	14%	58	32%	**19.32**	**<.001**
Footer menu option “Recipes”	45	25%	80	44%	**18.95**	**<.001**
Footer menu option “Plus”	23	13%	28	15%	.46	.50
Footer menu option “Plans”	37	20%	72	40%	**18.95**	**<.001**
Footer menu option “Settings”	26	14%	57	31%	**16.36**	**<.001**
Classic feature	70	39%	46	25%	**11.50**	**.001**
Simple rules icon	3	2%	9	5%	n/a	.07
Ketogenic Medium feature	101	56%	68	37%	**16.25**	**<.001**
5:2 feature	67	37%	52	29%	**4.00**	**.046**
Take the Test feature	108	59%	131	72%	**9.88**	**.002**

Note: n/a = chi square values are not applicable if fewer than 25 discordant pairs; binominal distributions are used for exact 2-tailed significance in these comparisons.

Appendix B. Survey items

Concept	Item	Response options
Feature expectation	Select all the elements you would expect to find in a health app. **Check all that apply.**	1 = Menu<br>2 = Search option<br>3 = Settings option<br>4 = Logo<br>5 = Log/Input data option<br>6 = Share with friends option<br>7 = Summary statistics<br>8 = Summary graph/chart<br>9 = Calendar<br>10 = Page title<br>11 = Login<br>12 = User profile
Feature Placement	Where do you think the **[feature]** should appear in the health app?<br><br>Select all the areas on the app screen below where you would expect the **[feature]** to appear. Selected areas will be shown in green.	Hot spots
App choice	Below are two health apps in development. Please review the apps and **select the app you would be mostly likely to download by checking the circle to the bottom left corner of the preview.** We will ask you questions about both, but first we want to know which one you prefer.	1 = Low prototypicality app<br>2 = High prototypicality app
Attention	For your selected app, what elements in the app caught your **attention**? Select all elements that grabbed your attention within the app preview below.	Hot spots
Predicted use	For your selected app, what elements in the app do you think you would **use**? Select all elements that you believe you would use within the app preview below.	Hot spots
Aesthetics: Simplicity	The layout appears too dense. (reverse coded)<br>The layout is easy to grasp.<br>Everything goes together on this app.<br>The app appears patchy. (reverse coded)<br>The layout appears well structured.	1 = Strongly disagree<br>2 = Somewhat disagree<br>3 = Neither agree nor disagree<br>4 = Somewhat agree<br>5 = Strongly agree
Aesthetics: Diversity	The layout is pleasantly varied.<br>The layout is inventive.<br>The design appears uninspired. (reverse coded)<br>The layout appears dynamic.<br>The design is uninteresting. (reverse coded)	1 = Strongly disagree<br>2 = Somewhat disagree<br>3 = Neither agree nor disagree<br>4 = Somewhat agree<br>5 = Strongly agree
Aesthetics: Colorfulness	The color composition is attractive.<br>The colors do not match. (reverse coded)<br>The choice of colors is botched. (reverse coded)<br>The colors are appealing.	1 = Strongly disagree<br>2 = Somewhat disagree<br>3 = Neither agree nor disagree<br>4 = Somewhat agree<br>5 = Strongly agree
Aesthetics: Craftsmanship	The layout appears professionally designed.<br>The layout is not up-to-date. (reverse)<br>The app is designed with care.<br>The design of the app lacks a concept. (reverse)	1 = Strongly disagree<br>2 = Somewhat disagree<br>3 = Neither agree nor disagree<br>4 = Somewhat agree

		5 = Strongly agree
Perceived ease of use	The app would be clear and understandable.<br>Getting the app to function would not require much mental effort.<br>I would find the app easy to use.	1 = Strongly disagree<br>2 = Somewhat disagree<br>3 = Neither agree nor disagree<br>4 = Somewhat agree<br>5 = Strongly agree
Perceived usefulness	Using the app would improve my health.<br>Using the app would make me more likely to meet my health goals.<br>I would find the app useful for achieving my health goals.	1 = Strongly disagree<br>2 = Somewhat disagree<br>3 = Neither agree nor disagree<br>4 = Somewhat agree<br>5 = Strongly agree
Intention to use	**[Randomized in construct matrix.]**<br>If the app were available, I would intend to use it in the next month.<br>If the app were available, I predict I would use it in the next month.	1 = Strongly disagree<br>2 = Somewhat disagree<br>3 = Neither agree nor disagree<br>4 = Somewhat agree<br>5 = Strongly agree
Perceived affordances	This app would allow me to…<br>Please check all that apply.	1 = Set health goals<br>2 = Track my progress<br>3 = Create new health habits<br>4 = Learn health tips<br>5 = Interact with others<br>6 = Earn rewards<br>7 = Make meeting my health goals easier<br>8 = Improve my health<br>9 = Increase my control over my health<br>10 = Have fun with technology<br>11 = Give me more information about my health<br>12 = Share my health data with friends<br>13 = Share my health data with a healthcare provider
Age	How old are you?	
Gender	How do you describe your gender identity?	1=Male<br>2=Female<br>3=Other __________
Hispanic	Are you of Hispanic, Latino, or Spanish origin?	1=Yes<br>0=No
Race	Which one of these groups would you say best represents your race?	1 = White<br>2 = Black or African American<br>3 = American Indian or Alaska Native<br>4 = Asian, or<br>5 = Pacific Islander<br>6 = Multicultural<br>7=Other
Education	What is the highest degree or level of school you have completed?	1 = Some high school<br>2 = High school diploma or GED<br>3 = Some college, no degree<br>4 = Associate degree<br>5 = Bachelor's degree<br>6= Masters degree (e.g., MA, MS)

		7 = Professional or Doctorate degree (e.g., JD, MD, PhD)
Health status	In general, would you say your health is …	1=Very Poor<br>2=Poor<br>3=Fair<br>4=Good<br>5=Very Good
Mental health status	In general, would you say your mental health is…	1=Very Poor<br>2=Poor<br>3=Fair<br>4=Good<br>5=Very Good
Health app use	Do you use a health app?	1=Yes<br>2=No
